# Machine Learning Prediction of Progression to Dialysis in Patients With Polycystic Kidney Disease: Population-Based Retrospective Cohort Study

**DOI:** 10.2196/80343

**Published:** 2026-03-16

**Authors:** Cheng-Hao Chang, Mingchih Chen, Ming-Hsien Tsai, Yen-Chun Huang, Hung-Hsiang Liou, Ben-Chang Shia, Chingying Liang, Yu-Wei Fang

**Affiliations:** 1 Division of Nephrology, Department of Internal Medicine, Shin-Kong Wu Ho-Su Memorial Hospital, No.95, Wen-Chang Road, Shih-lin District, Taipei, 111, Taiwan, 886-912376017; 2 Graduate Institute of Business Administration, College of Management, Fu Jen Catholic University, New Taipei City, Taiwan; 3 AI Development Center, Fu Jen Catholic University, New Taipei City, Taiwan; 4 Department of Medicine, Fu Jen Catholic University School of Medicine, New Taipei City, Taiwan; 5 Department of Artificial Intelligence, Tamkang University, New Taipei City, Taiwan; 6 Division of Nephrology, Department of Internal Medicine, Hsin-Jen Hospital, New Taipei City, Taiwan

**Keywords:** autosomal dominant polycystic kidney disease, ADPKD, artificial intelligence, machine learning, dialysis risk, predictive modeling

## Abstract

**Background:**

Autosomal dominant polycystic kidney disease (ADPKD), characterized by progressive cyst growth and renal decline, is the leading genetic cause of end‐stage renal disease.

**Objective:**

This study aims to develop and validate machine learning (ML) models for predicting the risk of progression to dialysis in patients with ADPKD using a nationwide administrative database. Early identification of high-risk patients is critical for timely monitoring.

**Methods:**

This retrospective cohort study used data from Taiwan’s National Health Insurance Research Database (2007‐2018) to identify newly diagnosed patients with ADPKD. Six ML algorithms, including logistic regression, random forest, and extreme gradient boosting (XGBoost), were employed to predict progression to dialysis. Models were developed using 10-fold cross-validation, with the Synthetic Minority Oversampling Technique applied within training folds to address class imbalance. An ensemble-based feature selection strategy was implemented to identify the most robust predictors and optimize final model performance. Model evaluation was conducted using a strict temporal split.

**Results:**

The study included 1856 patients with ADPKD, of whom 302 (16.27%) progressed to dialysis. Multivariable Cox regression identified several significant risk factors, including age 66 years and older (hazard ratio [HR] 4.63, 95% CI 2.71‐7.92; *P*<.001), anemia (HR 4.33, 95% CI 3.25‐5.78; *P*<.001), congestive heart failure (HR 1.81, 95% CI 1.29‐2.54; *P*<.001), and acute kidney injury (HR 1.69, 95% CI 1.19‐2.41; *P*=.003). Among the ML models, the XGBoost model, using an optimized set of 27 features, demonstrated the highest predictive performance on the held-out temporal test set (accuracy 98.3%; area under the curve 0.955; *F*_1_-score 0.800; Brier score 0.022). The top predictors in the XGBoost model largely aligned with age, comorbidity burden, anemia, and cardiovascular disease markers. Medication use (eg, anticoagulants, loop diuretics, febuxostat) was also among the most influential predictors; however, medication-related predictors should be interpreted as proxies for disease complexity rather than direct risk modulators.

**Conclusions:**

ML models can predict dialysis risk in patients with ADPKD using administrative data with temporal validation. This approach may support risk stratification by helping identify individuals at higher predicted risk who may warrant closer monitoring and further specialist evaluation.

## Introduction

### Background

Polycystic kidney disease (PKD) is the leading genetic cause of end-stage renal disease (ESRD). The most common mutations in PKD occur in the *PKD1* or *PKD2* gene, which encode the proteins polycystin-1 and polycystin-2, respectively. These mutations result in abnormal cell proliferation, fluid secretion, and differentiation, leading to the formation and growth of cysts [1]. The progressive nature of renal cysts may cause structural and functional changes in the kidney. According to the latest guidelines, the estimated prevalence of PKD varies widely, ranging from approximately 2 to 14 per 10,000 individuals, depending on the study methodology and population characteristics [2].

Contemporary management of autosomal dominant polycystic kidney disease (ADPKD) has evolved to include both the management of associated complications and direct interventions aimed at the disease mechanism. A cornerstone of care remains the control of hypertension, a common and impactful complication. Angiotensin-converting enzyme inhibitors (ACEIs) and angiotensin II receptor blockers (ARBs) are widely recommended for their established efficacy in blood pressure control and proteinuria mitigation [3,4]. In addition, lifestyle interventions, such as dietary sodium restriction and weight management, play a crucial role in preserving renal function [5,6]. In parallel, disease-modifying therapies, such as the vasopressin V2 receptor antagonist tolvaptan, have been developed to slow cyst growth in select patients with rapidly progressing disease, who are often identified using prognostic markers such as genetic status or total kidney volume (TKV) [7]. Furthermore, other medications, such as statins and metformin, are frequently prescribed to manage concurrent conditions in this population, though their role as primary treatment options for ADPKD itself remains under investigation [8,9].

### Study Objective

While risk stratification using advanced tools is crucial for guiding specialized treatments, a significant need remains for risk assessment methods based on accessible, routinely collected data. Currently, the gold-standard models for prognostication in ADPKD include the Mayo Imaging Classification, which utilizes height-adjusted TKV and patient age to predict the rate of estimated glomerular filtration rate (eGFR) decline, and the PROPKD score, which integrates genetic information (*PKD1* vs *PKD2* mutation type) with early clinical manifestations [2,10,11]. Although these models are invaluable, their broad implementation is often hindered by their dependence on specialized and resource-intensive evaluations. For instance, the Mayo Classification requires magnetic resonance imaging (MRI) or computed tomography to measure TKV, while the PROPKD score necessitates comprehensive genetic sequencing. These resources can be costly and are not universally available in all clinical settings, creating a gap for more scalable screening tools.

In parallel with these clinical tools, artificial intelligence (AI) has been increasingly used in ADPKD management. AI models have been developed to predict glomerular filtration rate decline, aiding in the early identification of high-risk patients and facilitating proactive management [12]. In imaging-based classifications, deep learning algorithms have been applied to automate ADPKD severity assessment, enhancing objectivity and reducing interobserver variability [13]. AI has also been instrumental in TKV quantification, a crucial prognostic marker in ADPKD. Deep learning-based segmentation models have demonstrated high accuracy in automated kidney segmentation from MRI and computed tomography images, significantly reducing manual workload [14,15]. While these advancements improve disease monitoring, they often still rely on the availability of imaging or detailed clinical data, leaving a gap for predictive models that can leverage more widely accessible administrative data to forecast hard clinical end points.

Therefore, this study aimed to develop and validate an AI-assisted model to identify significant predictors of progression to dialysis in patients with ADPKD using a nationwide administrative database. We evaluated multiple machine learning (ML) algorithms using a prespecified temporal split for model development and evaluation and summarized predictors to enhance interpretability and clinical usability. Our goal was to create a complementary tool for risk stratification, potentially aiding clinicians in monitoring patients and identifying those who may warrant more specialized evaluation.

## Methods

### Data Sources

This retrospective cohort study was based on the National Health Insurance Research Database (NHIRD), which is derived from Taiwan’s universal compulsory health insurance system covering nearly 99% of the 23 million individuals in Taiwan since 1998 [16]. The database includes patients’ hospitalization and outpatient visit records, and each individual was continuously followed. Disease diagnoses were coded according to the International Classification of Diseases, Ninth Revision, Clinical Modification (*ICD-9*-CM). The NHI Administration fully adopted *ICD-10*-CM for clinical records in 2016.

### Ethical Considerations

This study was conducted in accordance with the principles of the Declaration of Helsinki and was reviewed and approved by the Ethics Review Board of Shin-Kong Wu Ho-Su Memorial Hospital (approval number 20211008R). The requirement for informed consent was waived by the review board because this study involved a secondary analysis of routinely collected administrative data, and no direct contact with individual participants occurred. The original data collection procedures and this secondary analysis were approved without the need for additional consent.

All data obtained from the NHIRD were fully deidentified prior to analysis. Personal identifiers were removed by the data holder, and the authors had no access to information that could be used to identify individual patients. Data access and analysis were conducted in compliance with relevant data protection regulations.

No financial or other compensation was provided to participants, as this study did not involve direct recruitment or interaction with human subjects.

The manuscript and all supplementary materials did not include any images or information that could lead to the identification of individual participants.

### Study Design and Population

 We identified new-onset ADPKD cases from the Catastrophic Illness Patient Registry between 2007 and 2018. To minimize potential misclassification bias from ICD coding, we implemented stringent validation criteria for both the primary diagnosis and comorbidities. The identification of patients with ADPKD followed a 2-step process: subjects were first identified using ICD diagnostic codes, and their diagnosis was then validated against Taiwan’s Catastrophic Illness Patient Registry. A catastrophic illness certificate requires formal review and approval by physicians, ensuring a high degree of diagnostic accuracy for our study cohort [17-19].

The index date was defined as the first date on which a patient met the ADPKD case definition and had validation in the Catastrophic Illness Patient Registry. Baseline predictors were ascertained during the 365-day lookback period preceding the index date.

To construct a clean incident cohort and reduce reverse causation, we excluded individuals aged below 19 years or above 85 years, those with missing core administrative information (eg, sex, age, or index date), and those with evidence of ADPKD during the 2005 to 2006 washout period. We also excluded patients with any evidence of dialysis (hemodialysis or peritoneal dialysis) before the index date and those with kidney transplantation before the index date. Patients were followed from the index date until initiation of dialysis, death, or December 31, 2019, whichever occurred first.

### Baseline Variables

The dataset includes a variety of demographic, clinical, and medication-related variables for individuals with ADPKD. The key demographic variables include sex and age. Clinical variables encompass a variety of diseases grouped as cerebrovascular (intracranial aneurysms, ischemic stroke, and hemorrhagic stroke), cardiovascular (hypertension, atrial fibrillation, congestive heart failure [CHF], peripheral vascular disease, arrhythmia, and ischemic heart disease), gastrointestinal (liver cirrhosis, peptic ulcer, diverticulosis, cholangitis, and acute pancreatitis), renal and urological (urinary tract infection [UTI] and acute kidney injury [AKI]), pulmonary (chronic obstructive pulmonary disease [COPD], pneumonia, and asthma), metabolic (dyslipidemia, diabetes mellitus, and gout), mental (dementia, anxiety, and depression), and other (glaucoma and anemia). Each comorbidity was confirmed if the corresponding diagnosis code appeared in at least 1 hospitalization record or at least 3 outpatient visit records within the 365-day lookback period preceding the index date.

Medication variables cover a broad spectrum, including antihypertensives (beta-blockers, alpha-blockers, ACEIs/ARBs, calcium channel blockers [CCBs], methyldopa, hydralazine, minoxidil, clonidine, potassium-sparing diuretics, thiazide diuretics, and loop diuretics), antidiabetic drugs (metformin, sulfonylureas, dipeptidyl peptidase-4 inhibitors, thiazolidinediones, other oral hypoglycemic agents, rapid-acting insulins, and long-acting insulins), antimetabolic agents (statins, fenofibrate, and other lipid-lowering agents), urate-lowering agents (benzbromarone, allopurinol, febuxostat), and other medications (nonsteroidal anti-inflammatory drugs [NSAIDs], sedative hypnotics, tranexamic acid, mammalian target of rapamycin inhibitors, sodium bicarbonate, anticoagulants, antiplatelet agents, and vitamin K). As with the definition of comorbidities, we adopted a widely used and validated approach to enhance specificity. Medication variables were quantified as the cumulative number of prescription days during the 365-day lookback period preceding the index date. This approach was used to capture exposure intensity beyond a binary indicator. The specific codes used to define all comorbidities and medications are detailed in Tables S1 and S2 in Multimedia Appendix 1, respectively.

### Outcome Definition and Follow-Up

The primary end point was ESRD, defined as initiation of dialysis or receipt of kidney transplantation without prior dialysis. Patients were followed from the index date until ESRD, death, or December 31, 2019, whichever occurred first. For the ML classification models, the binary outcome was ESRD by the end of follow-up (event=1). In the ML analysis, the label reflects whether ESRD was observed by December 31, 2019, and does not represent a fixed-horizon risk. Patients without ESRD were labeled as nonevents (event=0), including those who died before reaching ESRD.

### Data Preprocessing and Handling of Missing Data

Our strategy for handling missing data involved 2 stages. First, case-wise deletion was performed to exclude any patient records with missing core administrative data (eg, sex, age, or date of diagnosis), as indicated in the study flowchart. For the final cohort, predictor variables were constructed such that no missing values remained. The absence of a record for a specific variable was treated as meaningful data rather than as a missing value. For instance, if a patient had no prescriptions for a given medication, the cumulative exposure days were coded as 0. Similarly, if a patient did not meet the predefined criteria for a comorbidity, the variable was coded as 0, indicating absence.

### Statistical Analysis

Categorical variables were presented as frequencies with percentages and compared using the chi-square test. Continuous variables were expressed as means with SDs and compared using the *t* test. Multivariable Cox proportional hazards regression models were used to estimate adjusted hazard ratios (HRs) and 95% CIs for the risk of dialysis initiation. A 2-tailed *P* value of <.05 was considered statistically significant. All statistical analyses were performed using SAS version 9.4 (SAS Institute Inc).

### ML Model Development and Feature Selection

Given the extended accrual period, we used a temporal split to preserve chronological separation between model development and evaluation. Records from 2007 to 2016 (80%) were used for model training, and records from 2017 to 2018 (20%) were reserved as an independent held-out test set for performance assessment.

Within the training set, we performed 10-fold cross-validation to improve robustness and reduce overfitting. To address class imbalance, Synthetic Minority Over-sampling Technique (SMOTE) was applied only within the training folds of each cross-validation iteration to avoid information leakage into the validation folds. Model performance was evaluated on the held-out test set using accuracy, Cohen κ, sensitivity, specificity, area under the curve (AUC), *F*_1_ score, and Brier score, recognizing that multiple complementary metrics are needed under imbalanced outcome distributions.

The 6 ML algorithms were chosen to encompass a broad spectrum of modeling techniques, ranging from interpretable linear models to complex, high-performance ensembles. Logistic regression was selected as a conventional statistical method widely used in clinical research, serving as a robust and interpretable baseline for comparison. To capture complex nonlinear relationships, we included 2 distinct models: classification and regression trees, a foundational decision tree algorithm, and multivariate adaptive regression splines, a flexible model adept at identifying interactions between predictors. Finally, to leverage the superior predictive power of ensemble learning, 3 state-of-the-art algorithms were selected [20]. Random forest represents the bagging method, which builds independent trees to reduce variance, whereas CatBoost and eXtreme Gradient Boosting (XGBoost) are representatives of boosting methods that construct models sequentially to correct the errors of their predecessors and often yield high accuracy. This comprehensive selection allowed for a thorough evaluation of predictive performance across different algorithmic families, and these methods have been successfully applied in similar medical informatics contexts [21-23] (see Supplementary Methods in Multimedia Appendix 1 for detailed model descriptions). Hyperparameters for each model were optimized using random search within the training set under 10-fold cross-validation. The held-out temporal test set was used only for final performance evaluation. The specific hyperparameters for all models are detailed in Table S3 in Multimedia Appendix 1. All model construction was conducted using Python (version 3.9; Python Software Foundation).

We employed a 2-pronged feature selection approach to identify the most significant predictors. In the first approach, each model performed feature selection based on its own internal mechanism, and its performance was evaluated using this algorithm-specific feature set. In the second approach, we constructed an overall consensus ranking of predictors using rank aggregation across the 6 algorithms. We prespecified that the best-performing algorithm would be selected based on performance on the held-out temporal test set, considering discrimination, class-imbalance–sensitive metrics, and calibration. For the selected best-performing model, we additionally evaluated parsimony by sequentially adding predictors according to the consensus ordering and selecting the feature count that maximized test-set performance.

## Results

### Characteristics of the Study Population

**Figure 1.**
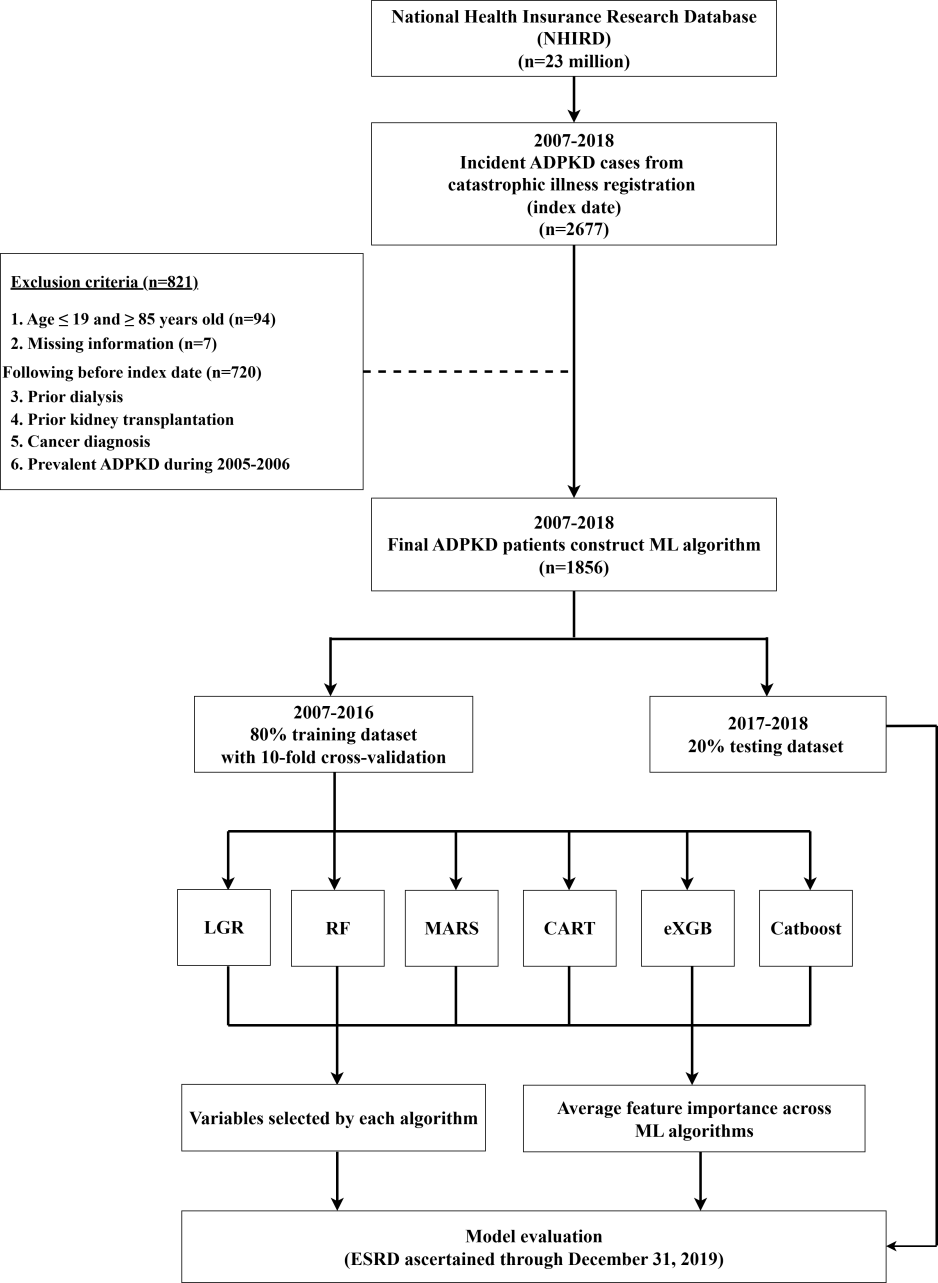
Study flowchart of patient selection and machine learning model development. The diagram shows the patient selection process from the NHIRD for the period 2007‐2018 and the subsequent workflow for model development. Patients were split temporally into a training set (2007‐2016) and a held-out test set (2017‐2018). ESRD was ascertained through December 31, 2019. ADPKD: autosomal dominant polycystic kidney disease; CART: classification and regression trees; ESRD: end-stage renal disease; eXGB: extreme gradient boosting; LGR: logistic regression; MARS: multivariate adaptive regression splines; ML: machine learning; RF: random forest.

From the NHIRD, 2677 incident ADPKD cases were identified between 2007 and 2018. After applying exclusion criteria (n=821), 1856 patients were included in the final cohort (Figure 1).

Table 1 summarizes baseline characteristics assessed during the 365-day lookback period preceding the index date. During follow-up through December 31, 2019, 302 patients (302/1856, 16.27%) initiated dialysis. Kidney transplantation without prior dialysis was observed in only 2 patients. Due to small cell size restrictions intended to protect patient privacy in the NHIRD data release process, transplantation events could not be analyzed separately; therefore, ESRD outcomes are presented primarily in terms of dialysis initiation. The remaining 1554 patients (1554/1856, 83.73%) did not initiate dialysis and were censored at death or end of follow-up.

**Table 1.** Baseline characteristics of the cohort stratified by dialysis initiation during follow-up.

Baseline characteristic	Autosomal dominant PKD^a^ (n=1856)	Dialysis initiation during follow-up	
		Yes	No
Sex , n (%)			
Male	1006 (54.20)	168 (55.63)	838 (53.93)
Female	850 (45.80)	134 (44.37)	716 (46.07)
Age group (y), n (%)			
≤40	687 (37.02)	56 (18.54)	631 (40.60)
41‐55	754 (40.63)	152 (50.33)	602 (38.74)
56‐65	265 (14.28)	56 (18.54)	209 (13.45)
≥66	150 (8.08)	38 (12.58)	112 (7.21)
Age, mean (SD)	45.85 (13.35)	50.94 (11.94)	44.86 (13.39)
CCI^b^ group, n (%)			
1‐2	1014 (54.63)	72 (3.88)	942 (50.75)
3‐5	660 (35.56)	168 (9.05)	492 (26.51)
6+	182 (9.81)	62 (3.34)	120 (6.47)
CCI scores, mean (SD)	2.58 (2.34)	4.18 (2.27)	2.27 (2.22)
Underlying disease, n (%)			
Cerebrovascular diseases			
Intracranial aneurysms	20 (1.08)	4 (1.32)	16 (1.03)
Ischemic stroke	51 (2.75)	11 (3.64)	40 (2.57)
Hemorrhagic stroke	91 (4.90)	27 (8.94)	64 (4.12)
Cardiovascular diseases			
Hypertension	1164 (62.72)	203 (67.22)	961 (61.84)
AF^c^	34 (1.83)	15 (4.97)	19 (1.22)
CHF^d^	178 (9.59)	77 (25.50)	101 (6.50)
PVD^e^	71 (3.83)	26 (8.61)	45 (2.90)
Arrhythmia	214 (11.53)	69 (22.85)	145 (9.33)
Ischemic heart disease	344 (18.53)	105 (34.77)	239 (15.38)
Gastrointestinal diseases			
Liver cirrhosis	33 (1.78)	14 (4.64)	19 (1.22)
Peptic ulcer bleeding	84 (4.53)	25 (8.28)	59 (3.80)
Diverticulosis	32 (1.72)	10 (3.31)	22 (1.42)
Cholangitis	39 (2.10)	16 (5.30)	23 (1.48)
Acute pancreatitis	41 (2.21)	20 (6.62)	21 (1.35)
Renal and urological diseases			
UTI^f^	938 (50.54)	196 (64.90)	742 (47.57)
AKI^g^	173 (9.32)	71 (23.51)	102 (6.56)
Lung diseases			
COPD^h^	319 (17.19)	71 (23.51)	248 (15.96)
Pneumonia	543 (29.26)	121 (40.07)	422 (27.16)
Asthma	207 (11.15)	31 (10.26)	176 (11.33)
Metabolic diseases			
Dyslipidemia	706 (38.04)	124 (41.06)	582 (37.45)
DM^i^	277 (14.92)	73 (24.17)	204 (13.13)
Gout	427 (23.01)	122 (40.40)	305 (19.63)
Mental diseases			
Dementia	47 (2.53)	15 (4.97)	32 (2.06)
Anxiety	321 (17.30)	62 (20.53)	259 (16.67)
Depression	250 (13.47)	46 (15.23)	204 (13.13)
Others			
Glaucoma	88 (4.74)	18 (5.96)	70 (4.50)
Anemia	461 (24.84)	199 (65.89)	262 (16.86)
Medication, mean days of use during the 365-day pre-index period (SD)			
Antihypertension			
Beta-blockers	95.85 (107.05)	92.24 (91.14)	97.15 (112.30)
Alpha-blockers	94.40 (106.66)	101.10 (107.30)	90.89 (106.30)
ACEI^j^/ARB^k^	160.11 (132.18)	130.10 (102.30)	167.30 (137.50)
CCB^l^	157.80 (136.36)	162.20 (122)	156.50 (140.40)
Methyldopa	26.79 (37.29)	17.97 (28.55)	31.61 (41.78)
Hydralazine	51.68 (65.70)	46.13 (60.99)	56.27 (69.50)
Minoxidil	53.04 (70.99)	43.11 (51.21)	68.30 (94.18)
Clonidine	41.69 (49.51)	42.35 (38)	40.92 (61.88)
Potassium-sparing diuretics	54.94 (83.58)	31.52 (68.85)	60.38 (85.97)
Thiazide diuretics	66.84 (83.42)	45.18 (67.33)	73.30 (86.68)
Loops diuretics	59.35 (94.95)	60.11 (84.12)	58.75 (102.70)
Antidiabetic			
Metformin	122.29 (122.92)	44.17 (43.31)	130.20 (125.70)
Sulfonylureas	130.89 (120.83)	103.20 (117.20)	137.30 (121.40)
DPP4i^m^	120.03 (101.88)	100.40 (103)	125 (101.50)
Other OHAs	92.69 (102.37)	113.50 (126.70)	82.84 (88.87)
Rapid-acting insulins	14.71 (49.95)	2.12 (8.94)	27.31 (68.23)
Long-acting insulins	100.09 (88.53)	130 (109.90)	84.26 (73.73)
Antimetabolic			
Statin	121.32 (111.87)	124.60 (118.10)	120.60 (110.50)
Fenofibrate	57.96 (72.62)	56.79 (81.12)	58.42 (69.38)
Other lipid-lowering agents	75.24 (90.44)	6.19 (2.38)	92.50 (93.65)
Urate-lowering agents			
Benzbromarone	65.45 (79.37)	41.72 (49.69)	71.38 (84.22)
Allopurinol	82.52 (97.02)	78.39 (84.91)	85.45 (105)
Febuxostat	104.04 (101.03)	92.67 (79.03)	108.20 (107.80)
Other			
NSAID^n^	15.76 (34.22)	15.69 (29.67)	15.78 (35.12)
Sedative hypnotics	92.03 (158.68)	118.70 (188.30)	83.81 (147.50)
Tranexamic acid	5.05 (16.89)	6.59 (11.14)	4.59 (18.24)
mTOR^o^ inhibitors	135.14 (148.87)	127.80 (140.60)	144 (174.70)
Sodium bicarbonate	9.50 (42.61)	20.52 (46.30)	7.25 (41.48)
Anticoagulants	110.46 (155.24)	113.10 (190.60)	109 (132.20)
Antiplatelet agents	211.22 (251.64)	204.20 (228.70)	214.30 (261.10)
Vitamin K	57.41 (91.58)	19.86 (43.90)	107.50 (114.30)

a PKD: polycystic kidney disease.

b CCI: Charlson Comorbidity Index.

c AF: atrial fibrillation.

d CHF: congestive heart failure.

e PVD: peripheral vascular disease.

f UTI: urinary tract infection.

g AKI: acute kidney injury.

h COPD: chronic obstructive pulmonary disease.

i DM: diabetes mellitus.

j ACEI: angiotensin-converting enzyme inhibitor.

k ARB: angiotensin II receptor blocker.

l CCB: calcium channel blocker.

m DPP4i: dipeptidyl peptidase-4 inhibitor.

n NSAID: nonsteroidal anti-inflammatory drug.

o mTOR: mechanistic target of rapamycin.

At baseline, the cohort had substantial cardiorenal comorbidity and medication use. The most prevalent comorbidities were hypertension (86.58%, 1606/1856) and UTI (50.38%, 935/1856), with pneumonia (29.26%, 543/1856), anemia (24.83%, 461/1856), and gout (23.01%, 427/1856) also common. Antihypertensive exposure was widespread (ACEI/ARB 161.30, SD 127.50 d; CCB 160.90, SD 133.70 d), and metabolic therapy was frequent (metformin 116.20, SD 137.10 d; long-acting insulin 91.71, SD 139.10 d).

Stratification by subsequent dialysis initiation highlighted clinically meaningful differences in baseline disease severity and management. Patients who later initiated dialysis were older (50.94, SD 11.94 vs 44.86, SD 13.39 y) and had higher comorbidity burden (Charlson Comorbidity Index [CCI] 4.18, SD 2.27 vs 2.27, SD 2.22; CCI≥6: 20.53% vs 7.72%), with the largest separations for anemia (65.89% vs 16.86%) and AKI (23.51% vs 6.56%). Baseline medication patterns were consistent with greater clinical complexity in the dialysis group, including lower metformin exposure (44.17, SD 95.41 vs 130.20, SD 148 d) and higher sodium bicarbonate exposure (20.52, SD 68.46 vs 7.25, SD 44.30 d).

### Risk Analysis for Progression to Dialysis

Table 2 presents the multivariable Cox regression results. After adjustment for sex, age category, CCI category, and comorbidities, male sex was associated with a higher risk of dialysis initiation (aHR 1.55, 95% CI 1.13‐2.11; *P*=.006). Age remained a strong predictor: compared with age younger than 55 years, the risk increased across categories. Among comorbidities, anemia showed the strongest association with dialysis initiation (aHR 4.33, 95% CI 3.25‐5.78; *P*<.001). Congestive heart failure, AKI, and gout were also associated with a higher risk. Hypertension, COPD, and anxiety were associated with lower hazard in the adjusted model.

**Table 2.** Risk for entering dialysis in the patients with ADPKD^a^.

	Cox model	
	aHR^b^ (95% CI)	*P* value
Sex		
Ref=Female	1	—^c^
Male	1.55 (1.13‐2.11)	.006
Age group		
Ref=<55	1	—
41‐55	3.01 (2.12‐4.30)	<.001
56‐65	4.13 (2.63‐6.50)	<.001
≥66	4.63 (2.71‐7.92)	<.001
CCI^d^ group		
Ref=12	1	—
345	1.42 (1.01‐1.98)	.04
6+	1.28 (0.74‐2.21)	.39
Underlying disease		
Cerebrovascular diseases		
Intracranial aneurysms	2.12 (0.71‐6.31)	.18
Ischemic stroke	1.22 (0.59‐2.49)	.59
Hemorrhagic stroke	1.03 (0.57‐1.86)	.91
Cardiovascular diseases		
Hypertension	0.70 (0.53‐0.94)	.02
AF^e^	0.66 (0.32‐1.32)	.24
CHF^f^	1.81 (1.29‐2.54)	<.001
PVD^g^	1.25 (0.79‐2.00)	.34
Arrhythmia	0.98 (0.69‐1.40)	.92
Gastrointestinal diseases		
Ischemic heart disease	1.09 (0.80‐1.48)	.58
Liver cirrhosis	0.91 (0.48‐1.72)	.77
Peptic ulcer bleeding	0.67 (0.42‐1.09)	.11
Diverticulosis	0.82 (0.38‐1.79)	.62
Cholangitis	1.10 (0.61‐1.96)	.76
Acute pancreatitis	1.18 (0.69‐2.04)	.55
Renal and urological diseases		
UTI^h^	0.84 (0.62‐1.14)	.26
AKI^i^	1.69 (1.19‐2.41)	.003
COPD^j^	0.61 (0.44‐0.86)	.005
Pneumonia	0.86 (0.65‐1.12)	.26
Asthma	0.74 (0.47‐1.15)	.18
Metabolic diseases		
Dyslipidemia	0.78 (0.51‐1.01)	.12
DM^k^	1.38 (0.97‐1.96)	.07
Gout	1.49 (1.08‐2.05)	.01
Mental diseases		
Dementia	0.71 (0.37‐1.34)	.29
Anxiety	0.53 (0.34‐0.82)	.004
Depression	1.33 (0.82‐2.16)	.26
Others		
Glaucoma	1.02 (0.60‐1.74)	.93
Anemia	4.33 (3.25‐5.78)	<.001

a ADPKD: autosomal dominant polycystic kidney disease.

b aHR: adjusted hazard ratio.

c Not applicable.

d CCI: Charlson Comorbidity Index.

e AF: atrial fibrillation.

f CHF: congestive heart failure.

g PVD: peripheral vascular disease.

h UTI: urinary tract infection.

i AKI: acute kidney injury.

j COPD: chronic obstructive pulmonary disease.

k DM: diabetes mellitus.

### Feature Importance Across ML Models

**Figure 2.**
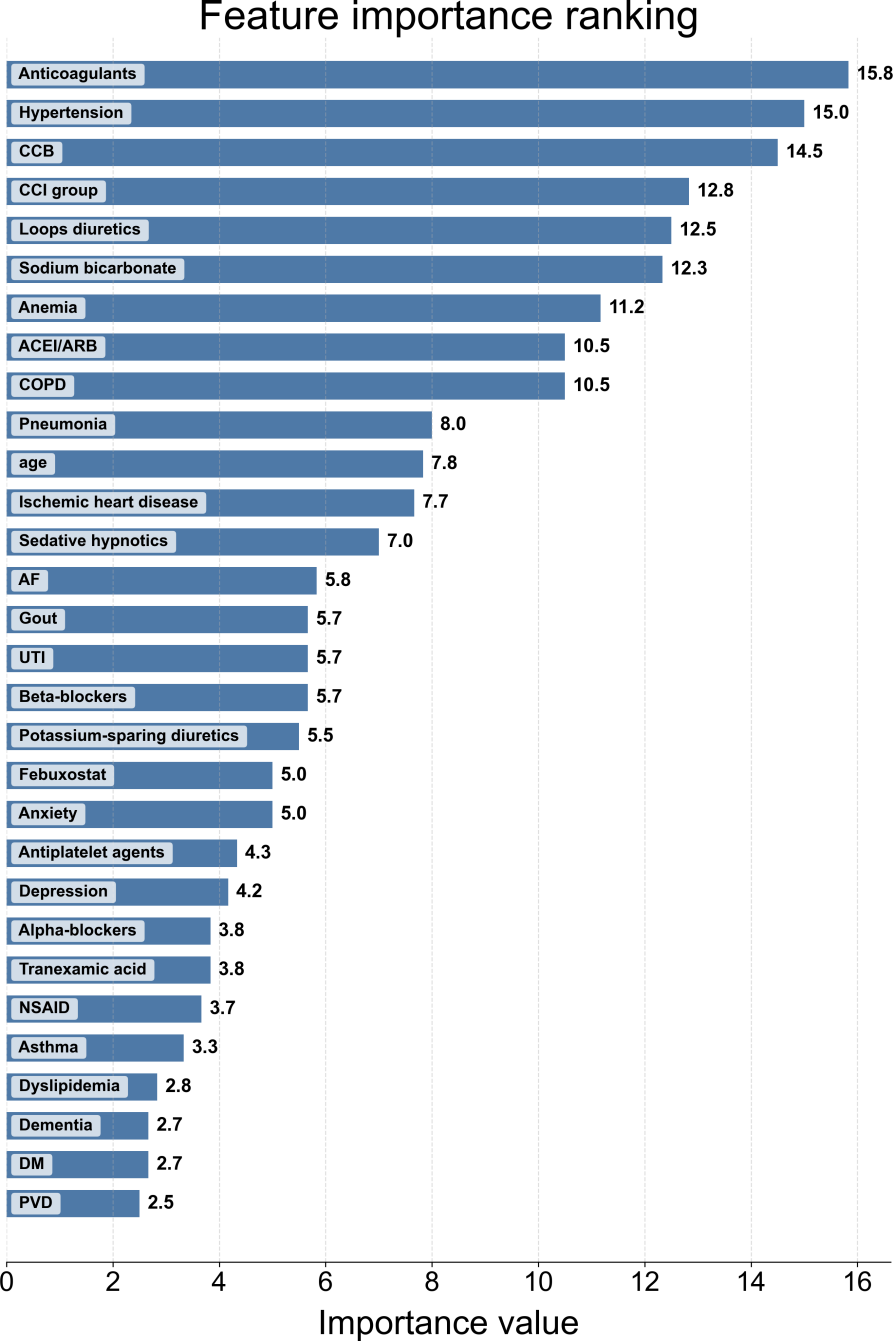
Overall consensus feature ranking aggregated across 6 machine learning algorithms. Predictors were ranked within each algorithm using the model’s native importance criterion; standardized ranks were then aggregated to generate an overall consensus ranking. ACEI: angiotensin-converting enzyme inhibitor; AF: atrial fibrillation; ARB: angiotensin II receptor blocker; CCB: calcium channel blocker; CCI: Charlson Comorbidity Index; COPD: chronic obstructive pulmonary disease; DM: diabetes mellitus; NSAID: nonsteroidal anti-inflammatory drug; PVD: peripheral vascular disease; UTI: urinary tract infection.

Figure 2 presents the relative importance of variables in predicting dialysis risk, as determined by the 6 ML models. A consensus emerged across the different algorithms, highlighting several key predictors. The top-ranked predictors spanned comorbidity burden and medication patterns, including anticoagulant use, hypertension, CCB use, higher CCI category, loop diuretics, sodium bicarbonate, anemia, and ACEI or ARB use, together with age and sex. Additional contributors included cardiorespiratory comorbidities, UTI, gout, and neuropsychiatric conditions and related medications.

### Performance Comparison of ML Models

Table 3 summarizes performance for 6 ML algorithms under 2 feature selection strategies: model-specific feature selection and a consensus feature set derived from rank aggregation. Using model-specific feature selection, XGBoost achieved the highest accuracy (0.9748) with specificity of 1, with an AUC of 0.9436 and a Brier score of 0.0254. Using the consensus feature set, performance improved across models. XGBoost achieved the highest accuracy (0.9832) and *F*_1_-score (0.8), whereas MARS achieved the highest AUC (0.9762) and CatBoost achieved the lowest Brier score (0.0217), indicating strong discrimination with favorable calibration across top-performing models.

**Table 3.** The model predictions with six different machine learning algorithms.

Methods	Accuracy	Kappa	Sensitivity	Specificity	AUC^a^	*F*_1_-score	Brier score
Variables selected by its own algorithm							
LGR^b^	0.9034	0.2552	0.4167	0.9292	0.7806	0.303	0.0771
RF^c^	0.9580	0.4790	0.4167	0.9867	0.9338	0.500	0.0352
MARS^d^	0.9664	0.6189	0.5833	0.9867	0.9558	0.6364	0.0260
CART^e^	0.9202	0.3037	0.4167	0.9469	0.9023	0.3448	0.0588
XGBoost^f^	0.9748	0.6551	0.5000	1	0.9436	0.6667	0.0254
CatBoost	0.9622	0.5521	0.5000	0.9867	0.9672	0.5714	0.0245
Variables selected based on the average results from six ML algorithms.							
LGR	0.9496	0.4735	0.5000	0.9735	0.9288	0.5000	0.0557
RF	0.9706	0.6174	0.5000	0.9956	0.9611	0.6316	0.0302
MARS	0.9790	0.7512	0.6667	0.9956	0.9762	0.7619	0.0232
CART	0.9454	0.4899	0.5833	0.9646	0.9399	0.5185	0.060
** **XGBoost	0.9832	0.7916	0.6667	1	0.9546	0.8000	0.0219
CatBoost	0.9790	0.7512	0.6567	0.9956	0.9676	0.7619	0.0217

a AUC: area under the curve.

b LGR: logistic regression.

c RF: random forest.

d MARS: multivariate adaptive regression splines.

e CART: classification and regression trees.

f XGBoost: extreme gradient boosting.

### Optimization of the XGBoost Model via Feature Selection

**Figure 3.**
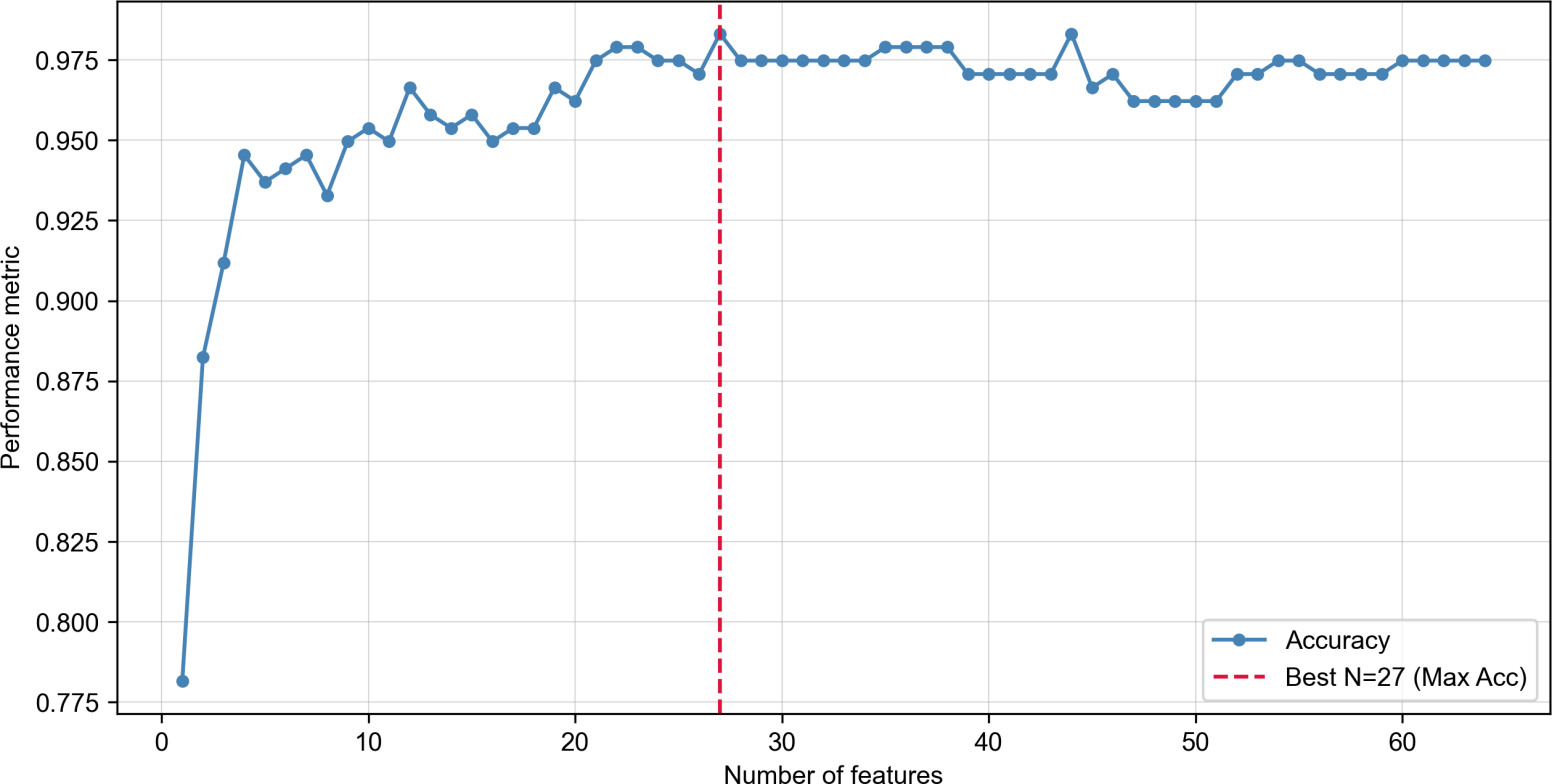
Optimization of extreme gradient boosting model performance by sequential feature addition. The plot shows the change in model accuracy (y-axis) as the number of top-ranked predictor variables (x-axis). The dashed vertical line indicates the point at which maximum accuracy was achieved with 27 variables.

Figure 3 shows the change in test set accuracy as top-ranked predictors from the consensus ranking were sequentially added to the XGBoost model. Accuracy increased rapidly as the highest-ranked predictors were included and reached its maximum with 27 predictors. This feature count was selected as the optimal balance between parsimony and predictive performance, and the 27-feature set was used for the final XGBoost model.

### Key Predictors in the Final XGBoost Model

The final XGBoost model used 27 baseline predictors selected during feature optimization (Table 4). This set included demographic and comorbidity burden measures (age, sex, and CCI category), major clinical conditions linked to renal risk or frailty (anemia, hypertension, ischemic heart disease, gout, pneumonia, COPD, UTI, anxiety, and depression), and multiple medication exposures. Key medication predictors included antihypertensive classes (ACEI or ARB, CCB, beta-blockers, and alpha-blockers), agents reflecting advanced renal management or metabolic status (loop diuretics, sodium bicarbonate, potassium-sparing diuretics, and rapid-acting insulin), and medications that may relate to underlying diseases (anticoagulants, antiplatelet agents, NSAIDs, sedative hypnotics, tranexamic acid, febuxostat, and statins). It is crucial to note that these medication-related predictors should be interpreted as proxies for underlying disease severity and complexity (ie, confounding by indication), rather than as direct causal risk factors.

**Table 4.** The variables selected for the best accuracy result in XGBoost. The rank is the average ranking from the average results of LGR^a^, RF^b^, MARS^c^, CART^d^, XGBoost^e^, and CatBoost.

Rank	Variable
1	Age
2	CCI^f^ scores
3	Anticoagulants
4	Loops diuretics
5	Alpha-blockers
6	NSAID^g^
7	Sex
8	CCB^h^
9	Sedative hypnotics
10	Anemia
11	ACEI^i^/ARB^j^
12	Hypertension
13	Gout
14	Tranexamic acid
15	Febuxostat
16	Ischemic heart disease
17	Statin
18	Antiplatelet agents
19	Beta-blockers
20	Pneumonia
21	Sodium bicarbonate
22	Potassium-sparing diuretics
23	Rapid-acting insulins
24	Anxiety
25	COPD^k^
26	Depression
27	UTI^l^

a LGR: logistic regression.

b RF: random forest.

c MARS: multivariate adaptive regression splines.

d CART: classification and regression trees.

e XGBoost: extreme gradient boosting.

f CCI: Charlson Comorbidity Index.

g NSAID: nonsteroidal anti-inflammatory drug.

h CCB: calcium channel blocker.

i ACEI: angiotensin-converting enzyme inhibitors.

j ARB: angiotensin receptor blocker.

k COPD: chronic obstructive pulmonary disease.

l UTI: urinary tract infection.

## Discussion

### Principal Findings

This retrospective cohort study used data from the NHIRD to predict ESRD progression in ADPKD using 6 ML algorithms, with ESRD operationalized as initiation of maintenance dialysis. On the held-out temporal test set, XGBoost demonstrated the best overall performance after feature optimization (accuracy 0.9832; *F*_1_ score 0.8). The final optimized XGBoost model was derived by sequentially adding top-ranked predictors and achieved peak accuracy with 27 variables. The most informative predictors reflected underlying disease severity and care intensity, including age, CCI, anemia, hypertension, ischemic heart disease, gout, pneumonia, COPD, UTI, anxiety, and depression, as well as medication exposures such as anticoagulants, loop diuretics, ACEI/ARB, CCB, and febuxostat.

### Interpretation of Major Clinical Risk Factors

Hypertension is a common early manifestation of ADPKD, often developing before renal function declines. It affects up to 86.6% of patients with ADPKD, frequently appearing around 30 years of age, with up to 20% of them diagnosed before the age of 20 [24,25]. The underlying mechanisms involve intrarenal ischemia, renin-angiotensin-aldosterone system (RAAS) activation, and endothelial dysfunction. Progressive cyst expansion leads to renal hypoperfusion and sustained RAAS stimulation, further exacerbating hypertension and accelerating kidney damage [26,27]. This mechanistic framework is consistent with our results, where hypertension and cardiovascular comorbidity markers, including ischemic heart disease and related medication patterns, contributed meaningfully to dialysis risk prediction.

Age and overall comorbidity burden were among the highest-ranked predictors in both the consensus feature ranking and the final XGBoost feature set. This pattern suggests that administrative claims-based models capture the cumulative burden of systemic illness, frailty, and health care utilization that often accompanies advanced kidney disease trajectories [28,29]. In our cohort, ischemic heart disease and cardiovascular medication proxies (eg, anticoagulants, antiplatelet agents, beta-blockers, and loop diuretics) were repeatedly selected, indicating that cardiovascular disease severity and its treatment intensity provide informative signals of near-term dialysis risk [30,31].

In our cohort, anemia showed the strongest association with dialysis initiation in the adjusted Cox model and was consistently selected by the ML algorithms. A study conducted in Japan found that, despite higher hemoglobin levels in ADPKD than in other CKD types, anemia remains a key predictor of renal disease progression. In nondialysis patients with ADPKD, lower hemoglobin levels were associated with faster kidney function decline, with men with hemoglobin levels less than 12 g/dL and women with hemoglobin levels less than 11 g/dL at higher risk [32]. Together, these findings support anemia as a clinically accessible marker of advanced disease and progression risk in ADPKD.

AKI and infection-related diagnoses such as pneumonia and UTI also contributed to the prediction. These events may accelerate loss of residual kidney function through hemodynamic instability, inflammatory injury, or nephrotoxic exposures, and they may also serve as markers of vulnerability and more frequent clinical encounters. In parallel, medication predictors such as sodium bicarbonate and NSAIDs plausibly reflect clinically recognized metabolic acidosis and nephrotoxic exposure that often accompany advanced CKD and precede dialysis initiation [33].

### Medication Predictors as Markers of Disease Severity

As highlighted earlier, a critical aspect of interpreting our findings is the potential for confounding by indication, particularly for medication-related predictors. In a retrospective cohort study, the use of a specific medication is often a marker of underlying disease severity rather than a cause of the outcome itself. Therefore, while these medications emerged as strong predictors in our model, they should be viewed primarily as indicators of a higher underlying risk profile, and our findings do not imply a causal relationship or recommend changes in prescribing practices. Consistent with prior work using administrative claims for kidney failure prediction, these medication patterns should be interpreted primarily as proxies for clinical complexity rather than as causal determinants of dialysis initiation [31].

Renin-angiotensin system inhibitors have been established as the first-line treatment due to their benefits in hypertension control [34]. In our model, the selection of additional antihypertensive classes (eg, alpha-blockers and CCB) and loop diuretics likely reflects difficult-to-control blood pressure, volume management needs, or concomitant cardiovascular disease rather than direct causal effects on renal decline. Similarly, anticoagulant and antiplatelet use may act as proxies for atrial fibrillation, vascular disease, or prior cardiovascular events, conditions that commonly cluster with advanced CKD and a higher risk of dialysis initiation.

Tranexamic acid was also selected among the final XGBoost predictors. In ADPKD, clinically significant gross hematuria is a common and clinically relevant event, and tranexamic acid has been used in small series for severe hematuria, although evidence remains limited [2,35]. Therefore, in claims-based modeling, tranexamic acid likely functions as a proxy for clinically significant hematuria or bleeding-related care that clusters with more advanced disease trajectories, rather than as a causal determinant of dialysis initiation.

Hyperuricemia is increasingly implicated in CKD progression, and our model identified gout and febuxostat as informative predictors of dialysis. This finding does not suggest a causal link but rather reflects prescribing patterns that align with both international guidelines and local reimbursement policies. Major clinical practice guidelines for ADPKD recommend allopurinol as the first-line urate-lowering therapy [2]. This standard is strictly reflected in Taiwan’s National Health Insurance system, where reimbursement for the second-line agent, febuxostat, was restricted during our study period. These restrictions likely limited its use primarily to patients with substantial preexisting conditions, most notably advanced chronic kidney disease (defined as eGFR <45 mL/min/1.73 m^2^) or other complications such as urate nephrolithiasis or severe tophaceous gout. Consequently, the predictive signal from febuxostat in our model arises because it serves as a powerful proxy, identifying a patient subgroup with higher baseline renal risk and greater disease complexity. Further prospective studies are needed to determine if urate-lowering therapy itself modifies the disease course in ADPKD.

Statins, primarily known for their cholesterol-lowering effects, also possess anti-inflammatory and antioxidant properties that could be beneficial in PKD. Chronic inflammation and oxidative stress play roles in the pathophysiology of PKD, contributing to cyst growth and renal function decline. Evidence regarding the efficacy of statins in PKD is equivocal. Some cohort studies and animal research suggest that statins may slow the decline in renal function in patients with PKD by reducing inflammation and oxidative stress [9]. However, findings from definitive clinical trials in PKD populations, which are scarce, remain inconclusive.

### Comparison With Existing Risk Stratification Tools

Our ML approach should be compared with existing clinical risk tools for ADPKD, such as the Mayo Imaging Classification and the PROPKD score. The primary advantage of our model lies in its broad accessibility. By leveraging nationwide administrative data, it bypasses the need for resource-intensive assessments, such as MRI for TKV measurement or genetic sequencing, making it a scalable and cost-effective tool for initial risk stratification across a large population. The main disadvantage, however, is the lack of direct pathophysiological data. Without TKV or genetic data, our model relies on surrogate markers of disease severity and cannot offer the same level of mechanistic insight or precision as the gold-standard tools. Therefore, our model should not be viewed as a replacement for these established methods but rather as a complementary instrument. Its optimal clinical role would be to serve as a first-line screening tool to efficiently identify high-risk patients who could then be prioritized for more definitive prognostic evaluations like imaging or genetic testing.

### Strengths and Limitations

To our knowledge, this study represents the first nationwide ML-based analysis of dialysis risk in ADPKD using administrative data. It provides a scalable framework for population-level risk stratification. The use of the NHIRD, a large population-based dataset with long-term follow-up, ensures high generalizability to the nationwide population of Taiwan.

Several limitations of this study should be acknowledged. First, its retrospective design precludes the establishment of causality. Second, and most importantly, our study is constrained by the nature of the NHIRD, which lacks the gold-standard prognostic markers for ADPKD including genetic data (PKD1/PKD2 status) and imaging biomarkers like TKV, and key laboratory values such as eGFR or proteinuria. Without direct access to TKV or genetic data, our model inherently relies on surrogate markers such as comorbidity burden and medication patterns to infer underlying risk. Despite the use of a washout period and validation via the Catastrophic Illness Patient Registry, truly incident ADPKD cases cannot be fully guaranteed in claims-based data. We therefore wish to emphasize that our model is proposed as a complementary tool for broad-based risk stratification, not as a replacement for comprehensive clinical evaluation. Third, because observation windows vary by index year, the binary labeling framework may under-ascertain events among patients with shorter follow-up and can bias absolute risk interpretation. In addition, calibration curves were not presented, limiting assessment of absolute risk calibration. Fourth, because deaths occurring before ESRD were labeled as nonevents in the binary ML framework, patients with high competing mortality may be misclassified as having low renal risk. Fifth, the measurement of medication exposure, quantified by prescription days, is a proxy that does not capture actual patient adherence, dosage, or treatment adjustments. This limits our ability to make definitive conclusions about the role of specific pharmacological agents. Finally, our model was developed using a Taiwanese population, and its performance and the relative importance of predictors may vary across different ethnic and geographic groups, which may limit generalizability.

### Future Work

Future studies should prioritize prospective validation in diverse external cohorts. Methodologically, fixed-horizon prediction and time-to-event approaches, including survival ML and competing-risk or multistate frameworks, should be evaluated to better align modeling with clinical trajectories. External validation datasets that include laboratory, genetic, and imaging biomarkers (eg, eGFR, proteinuria, PKD1/PKD2 status, and TKV) will enable integrated, multitiered risk stratification and more complete assessment of calibration and clinical utility. Moreover, instance-level explainability methods (eg, SHAP) can be explored to support clinical interpretability.

### Conclusions

This study developed and evaluated 6 ML models to classify dialysis initiation risk in a nationwide ADPKD cohort using administrative claims data and a prespecified temporal validation design. On the held-out temporal test set, XGBoost showed the best overall performance. The most informative predictors primarily reflected disease severity and care intensity captured in claims data, including age, comorbidity burden, anemia, and hypertension, whereas medication variables should be interpreted as noncausal proxies of clinical complexity.

## Supplementary material

10.2196/80343 Multimedia Appendix 1 Supplementary methods and tables, including model implementation details for the six machine learning algorithms, ICD-9-CM/ICD-10-CM codes for comorbidities (Table S1), ATC codes and drug lists (Table S2), and tuned hyperparameters with software packages and versions (Table S3).

## Abbreviations

ACEI: angiotensin-converting enzyme inhibitor
ADPKD: autosomal dominant polycystic kidney disease
AI: artificial intelligence
AKI: acute kidney injury
ARB: angiotensin II receptor blocker
AUC: area under the curve
CCB: calcium channel blocker
CCI: Charlson Comorbidity Index
COPD: chronic obstructive pulmonary disease
ESRD: end-stage renal disease
HR: hazard ratio
ML: machine learning
MRI: magnetic resonance imaging
NHIRD: National Health Insurance Research Database
NSAID: nonsteroidal anti-inflammatory drug
PKD: polycystic kidney disease
TKV: total kidney volume
UTI: urinary tract infection
XGBoost: extreme gradient boosting
