# Supplementary material for: Machine Learning Prediction of Progression to Dialysis in Patients With Polycystic Kidney Disease: Population-Based Retrospective Cohort Study
Source: JMIR Med Inform. 2026 Mar 16;14:e80343. doi: 10.2196/80343 (PMC12991194; doi:10.2196/80343)
Supplement: Multimedia Appendix 1 [file medinform-v14-e80343-s001.docx]

**Predicting the Risk of Progression to Dialysis in Patients with Polycystic Kidney Disease: A Population-based Machine Learning Study**

Cheng-Hao Chang^1*^, Mingchih Chen PhD^2,3*^, Ming-Hsien Tsai, MD, PhD^1,4^, Yen-Chun Huang PhD^5^, Hung-Hsiang Liou^6^, Ben-Chang Shia, MD, PhD^2,3^, Chingying Liang^2,3^, Yu-Wei Fang MD, PhD^1,4^

*Co-first author

^1^ Division of Nephrology, Department of Internal Medicine, Shin-Kong Wu Ho-Su Memorial Hospital, Taipei, Taiwan.

^2^ Graduate Institute of Business Administration, College of Management, Fu Jen Catholic University, New Taipei City, Taiwan.

^3^ AI Development Center, Fu Jen Catholic University, New Taipei City, Taiwan.

^4^ Department of Medicine, Fu Jen Catholic University School of Medicine, New Taipei City, Taiwan.

^5^ Department of Artificial Intelligence, Tamkang University, New Taipei City, Taiwan.

^6^ Division of Nephrology, Department of Internal Medicine, Hsin-Jen Hospital, New Taipei City, Taiwan.

**Running title:** Predicting Dialysis Risk in ADPKD with Machine Learning

***Reprints and correspondence:**

Address correspondence to Yu-Wei Fang, M.D. PhD

Division of Nephrology, Department of Internal Medicine,

Shin-Kong Wu Ho-Su Memorial Hospital, Taipei, Taiwan, ROC.

E-mail: [m005916@gmail.com](mailto:m005916@gmail.com)

**Supplementary Online Content**

[**Supplementary Method** 4](#_Toc221986300)

[**Supplementary Table S1: Code for comorbidities** 8](#_Toc221986301)

[**Supplementary Table S2: Code for drugs** 10](#_Toc221986302)

[**Supplementary Table S3: Hyperparameters Used for Machine Learning Models** 13](#_Toc221986303)

**Supplementary Method**

**Model implementation**

**LGR**

Logistic regression is a statistical technique utilized to assess the relationship between one or more independent variables and a binary dependent variable[1, 2]. This model can be adjusted to accommodate multiple independent variables, enabling a nuanced analysis of the distinct impact of each variable on the outcome[3]. A key benefit of logistic regression is that its coefficients can be interpreted as odds ratios, which aids in understanding the results more clearly. However, reliable inferences drawn from logistic regression are contingent upon the satisfaction of certain assumptions, such as the independence of observations and correct model specification[3, 4].

**CART**

In 1984, Breiman et al. presented the Classification and Regression Tree (CART) algorithm, which not only became a fundamental tool in statistical modeling and machine learning but also had a significant impact on the field of data analysis[5].

CART, recognized as a traditional decision tree methodology, autonomously identifies classification features and establishes node thresholds through specific algorithms [6]. It is capable of managing highly skewed or multimodal numerical data, which makes it particularly useful in intricate medical data analysis[7].

This algorithm generates a series of classification and regression rules through a recursive process to systematically evaluate the data. Specifically, the CART algorithm uses Gini impurity as a key metric to calculate the impurity of the data at each possible split, effectively guiding the process of variable splitting. It continuously divides the variables recursively, making decisions at each node until a predefined stopping condition is reached[2, 8]. When this condition is met, the growth of the tree stops, resulting in a final model that can be used for prediction or classification tasks. This method not only allows for the creation of complex decision structures but also maintains interpretability and ease of use. While CART is often seen as a classification algorithm, it does not ensure consistent classification performance across various scenarios. Consequently, we creatively utilize CART as a swift and effective feature selection algorithm in place of traditional classifiers.

**RF**

In 2001, Breiman introduced an advanced method to tackle challenges associated with decision trees (DT) [5, 9]. Random Forest (RF) classifiers build reliable classifications by aggregating multiple decision trees, making them particularly effective for high-dimensional data and identifying the most relevant variables for distinguishing target classes. It’s employs bagging and bootstrap aggregation principles, selecting a random subset of variables for node splitting during the construction of each CART tree [5, 10]. RF classifiers demonstrate strong performance in classification accuracy and stability across various settings, proving to be valuable tools for processing remote sensing data and holding significant potential for future research in the field.

**MARS**

Multivariate Adaptive Regression Splines (MARS) is a nonparametric statistical machine learning technique developed by Friedman et al. in 1991.[11]. This method constructs regression models automatically through recursive partitioning, enabling it to handle high-dimensional data with flexibility and to effectively capture intricate nonlinear relationships between independent and dependent variables. Unlike conventional regression methods, MARS does not impose any prior assumptions about these relationships. Instead, it utilizes basis functions to transform the data into a higher-dimensional space, enhancing predictive accuracy.

Overall, MARS serves as a powerful and adaptable tool for modeling complex data structures without the need for predefined relationship forms.[11, 12]. In summary, MARS provides a flexible and powerful tool for effectively modeling complex data.

**XGBoost**

XGBoost, developed by Chen and Guestrin, is an efficient and flexible machine learning algorithm widely used for classification and regression tasks[13]. It is based on the principle of gradient boosting trees, particularly suitable for handling large-scale datasets and preventing overfitting. Each new tree is designed to correct the prediction errors of the previous trees[14]. By integrating multiple learners, XGBoost attains greater accuracy compared to individual models. [8]. The main features of XGBoost include efficiency in accelerating the training process, regularization to control overfitting, support for various objective functions, and the ability to customize optimization objectives.

**CatBoost**

CatBoost, introduced by Prokhorenkova et al., is a gradient boosting decision tree algorithm designed for high predictive performance on tabular data, with native support for categorical features[15]. Similar to other boosting methods, CatBoost builds an ensemble of decision trees sequentially, where each new tree is trained to reduce the residual errors of the previous trees under a specified loss function[16]. A distinguishing feature of CatBoost is its treatment of categorical variables using ordered target statistics with permutation-driven training, which helps reduce target leakage and prediction shift compared with naive target encoding. CatBoost also uses symmetric (oblivious) trees and built-in regularization mechanisms that can improve training efficiency and generalization while minimizing the need for extensive categorical preprocessing. Key features of CatBoost therefore include native handling of categorical features, ordered boosting for improved robustness, efficient tree structures, and regularization to mitigate overfitting in practical prediction tasks.

**Reference**

1. Zhang, H., et al. *Improving prediction accuracy for logistic regression on imbalanced datasets*. in *2019 IEEE 43rd annual computer software and applications conference (COMPSAC)*. 2019. IEEE.

2. Huang, Y.-C., et al. *Machine-learning techniques for feature selection and prediction of mortality in elderly CABG patients*. in *Healthcare*. 2021. MDPI.

3. Schober, P. and T.R. Vetter, *Logistic regression in medical research.* Anesthesia & Analgesia, 2021. **132**(2): p. 365-366.

4. Huang, Y.-C., et al., *A framework to predict second primary lung cancer patients by using ensemble models.* Annals of Operations Research, 2023: p. 1-25.

5. Breiman, L., *Random forests.* Machine learning, 2001. **45**: p. 5-32.

6. Krzywinski, M. and N. Altman, *Classification and regression trees.* Nature Methods, 2017. **14**(8): p. 757-758.

7. Dong, N., et al., *Cervical cell classification based on the CART feature selection algorithm.* Journal of Ambient Intelligence and Humanized Computing, 2021. **12**: p. 1837-1849.

8. Wu, T.-E., et al., *Evaluating the effect of topical atropine use for myopia control on intraocular pressure by using machine learning.* Journal of Clinical Medicine, 2020. **10**(1): p. 111.

9. Gregorutti, B., B. Michel, and P. Saint-Pierre, *Grouped variable importance with random forests and application to multiple functional data analysis.* Computational Statistics & Data Analysis, 2015. **90**: p. 15-35.

10. Sun, Z., et al., *An improved random forest based on the classification accuracy and correlation measurement of decision trees.* Expert Systems with Applications, 2024. **237**: p. 121549.

11. Friedman, J.H., *Multivariate adaptive regression splines.* The annals of statistics, 1991. **19**(1): p. 1-67.

12. Ekman, T. and G. Kubin. *Nonlinear prediction of mobile radio channels: measurements and MARS model designs*. in *1999 IEEE international conference on acoustics, speech, and signal processing. proceedings. ICASSP99 (Cat. No. 99CH36258)*. 1999. IEEE.

13. Chen, T. and C. Guestrin. *Xgboost: A scalable tree boosting system*. in *Proceedings of the 22nd acm sigkdd international conference on knowledge discovery and data mining*. 2016.

14. Frifra, A., et al., *Harnessing LSTM and XGBoost algorithms for storm prediction.* Scientific Reports, 2024. **14**(1): p. 11381.

15. Prokhorenkova L, Gusev G, Vorobev A, Dorogush AV, Gulin A. CatBoost: unbiased boosting with categorical features. In: Advances in Neural Information Processing Systems (NeurIPS). 2018.

16. Friedman JH. Greedy function approximation: A gradient boosting machine. Annals of Statistics. 2001;29(5):1189-1232

**Supplementary Table S1: Code for comorbidities**

|  | ICD-9-CM | ICD-10-CM |
| --- | --- | --- |
| ***Comorbidities*** | | |
| Hypertension | 401 – 405 | I10-14 |
| Diabetes mellitus | 250 | E10-E14 |
| MI | 410, 412 | I21, I22, I24.1, I24.8, I24.9,I20.0, I25.2 |
| CHF | 428.0, 428.1, 428.9 | I50 |
| AF | 427.31 | I48 |
| PVD | 250.7, 440.2-3,440.8-9,  443,444.22,444.8,447.8-9 | E08.51-52, E08.59, E09.51-52, E09.59, E10.51-52, E10.59, E11.51-52, E11.59, E13.51-52, E13.59, I70.2-I70.9, I73, I74.2-I74.9, I75.011-I75.029, I75.89, I77.3, I77.89, I77.9, I79.1, I79.8 |
| Stroke | 430–438 | I60- I63, I65-I69, G45-G46 |
| Hyperlipidemia | 272.0–272.4 | E78 |
| COPD | 490–492, 496 | J40-J44, J47 |
| Hyperuricemia | 790.6 | E79.0 |
| Gout | 274.0, 274.1X, 274.8X, 274.9 | M10 |
| Liver cirrhosis | 571.5 | K74, K74.1, K74.2, K74.60, K74.69 |
| **Abbreviation:**  ICD, international classification of disease; MI, myocardial infarct; CHF, congestive heart failure; AF, atrial fibrillation; PVD, peripheral vascular disease; COPD, chronic obstructive pulmonary disease; | | |

**Supplementary Table S2: Code for drugs**

| **Drug type** | **ATC classification system codes** | **Drug name** |
| --- | --- | --- |
| Angiotensin-converting enzyme inhibitors / angiotensin receptor blockers | C09A, C09B, C09C, C09D | Captopril, Enalapril, Lisinopril, Perindopril, Ramipril, Quinapril, Benazepril, Cilazapril, Fosinopril, Imidapril, Losartan, Eprosartan, Valsartan, Irbesartan, Candesartan, Telmisartan, Olmesartan, Azilsartan |
| Beta blocking agents | C07A, C07B, C07CA03, C07DA06 | Alprenolol, Oxprenolol, Pindolol, Propranolol, Timolol, Sotalol, Nadolol, Carteolol, Bupranolol, Metoprolol, Atenolol, Acebutolol, Betaxolol, Bevantolol, Bisoprolol, Esmolol, Nebivolol, Labetalol, Carvedilol |
| Calcium channel blockers | C08C, C08D, C08E, C09BB, C09DB,  C09DX,C10BX | Amlopidine, Felodipine, Isradipine, Nicardipine, Nifedipine, Nimodipine, Nisoldipine, Nitrendipine, Lacidipine, Barnidipine, Lercanidipine, Cilnidipine, Benidipine, Verapamil, Diltiazem |
| Diuretic | C03AA, C03BA, C03CA, C03CC, C03DA, C03DB, C03EA, C09DX | bendroflumethiazide, hydrochlorothiazide, trichlormethiazide,  cyclopenthiazide, Benzylhydrochlorothiazide, chlortalidone, metolazone, indapamide, furosemide, bumetanide, torasemide,  etacrynic acid, potassium canrenoate, spironolactone, eplerenone,  amiloride, triamterene |
| Insulin | A10AB, A10AC,A10AD,A10AE | Insulin(human), insulin (pork), insulin lispro, insulin aspart, insulin glulisine, insulin glargine, insulin detemir, insulin degludec, insulin glargine and lixisenatide |
| Lipid-lowering agents | C10AA, C10AB, C10AC, C10AD, C10AX, C10BA, C10BX03 | Simvastatin, Lovastatin, Pravastatin, Fluvastatin, Atorvastatin, Rosuvastatin, Pitavastatin, Clofibrate, Bezafibrate, aluminium clofibrate, Gemfibrozil, Fenofibrate, Simfibrate, Etofibrate,  Cholestyramine, Colestipol, Dextran sulfate sodium, Niceritrol,  Niacin, Nicofuranose, Acipimox, Nicomol, Soysterol (=Esterol),  Probucol, ezetimibe, evolocumab, alirocumab |
| Oral hypoglycemic agent | A10BB, A10BD09, A10BF01, A10BG, A10BH, A10BX02, A10BX03 | Glibenclamide, Chlorpropamide, Tolbutamide, Glibornuride, Tolazamide, Glipizide, Gliquidone, Gliclazide, Glimepiride, Acetohexamide, Rosiglitazone, Pioglitazone, Sitagliptin, Vildagliptin, Saxagliptin, Alogliptin, Linagliptin, Repaglinide,  Nateglinide |
| Benzodiazepines | N03AE, N05BA, N05CD, N05CF | Clonazepam, Diazepam, Chlordiazepoxide, Medazepam, Oxazepam, Clorazepate, Lorazepam, Bromazepam, Clobazam, Prazepam, Alprazolam, Nordazepam, Fludiazepam, Cloxazolam, Oxazolam, Flurazepam, Nitrazepam, Flunitrazepam, Estazolam, Triazolam, Lormetazepam, Temazepam, Midazolam, Brotizolam, Nimetazepam, Zopiclone, Zolpidem, Zaleplon, Eszopiclone |
| Uric acid lowering agent | M04AA, M04AB, M04AC | Allopurinol, Febuxostat, Probenecid, Sulfinpyrazone, Benzbromarone, Colchicine |
| Anti-coagulant | B01AA, B01AC, B01AE, B01AF | phenindione, warfarin, clopidogrel, iclopidine, acetylsalicylic acid,  dipyridamole, epoprostenol, iloprost, abciximab, eptifibatide, tirofiban, treprostinil, prasugrel, cilostazol, ticagrelor, selexipag,  combinations, Cilostazol, dabigatran etexilate, rivaroxaban, apixaban, edoxaban |
| Non-Steroidal Anti-Inflammatory Drug | M01AB, M01AE, M01AG, M01AX, M01AC, M01AH, M01AX01, M01AX17 | Indomethacin, sulindac, tolmetin, diclofenac, alclofenac, etodolac, acemetacin, ketorolac, aceclofenac, ibuprofen, naproxen, ketoprofen, fenoprofen, fenbufen,  flurbiprofen, tiaprofenic acid, alminoprofen,  mefenamic acid, tolfenamic acid, flufenamic acid,  meclofenamic acid, niflumic acid, benzydamine,  tiaramide Hcl, piroxicam, piroxicam, tenoxicam, meloxicam, celecoxib, rofecoxib, etoricoxib, nabumetone, nimesulide |
| Abbreviation: ATC, anatomical therapeutic chemical; | | |

**Supplementary Table S3: Hyperparameters Used for Machine Learning Models**

Note: Hyperparameters were tuned via Random Search on the training set only (n_iter = 100) using 10-fold cross-validation. The best configuration by mean cross-validated AUC was selected. The held-out temporal test set was not used for tuning or model selection.

| **Algorithm** | **Implementation (package, version)** | **Tuned hyperparameters (best params)** |
| --- | --- | --- |
| LGR | scikit-learn 1.8.0 | solver=liblinear;  penalty=l2;  C=100 |
| RF | scikit-learn 1.8.0 | n_estimators=300;  min_samples_split=2;  max_depth=None |
| MARS | sklearn-contrib-py-earth 0.1.0 | max_degree=2;  penalty=3 |
| CART | scikit-learn 1.8.0 | max_depth=7;  ccp_alpha=0.01;  min_samples_leaf=50 |
| XGBoost | xgboost 3.1.3 | n_estimators=100;  max_depth=5;  learning_rate=0.1 |
| CatBoost | catboost 1.2.8 | learning_rate=0.1;  iterations=200;  depth=6 |
